# Supplementary material for: Comparative multiomics analysis of cell physiological state after culture in a basket bioreactor
Source: Sci Rep. 2022 Nov 23;12:20161. doi: 10.1038/s41598-022-24687-4 (PMC9686226; doi:10.1038/s41598-022-24687-4)
Supplement: Supplementary file 1 — Supplementary Information 1. [file 41598_2022_24687_MOESM1_ESM.zip › raw data/Transcriptomic raw data/6.Enrichment/1.GO/TE1vsTC1/GSEA/TE1/GROWTH_FACTOR_ACTIVITY/GROWTH_FACTOR_ACTIVITY(GO_0008083).html]

Details for gene set GROWTH\_FACTOR\_ACTIVITY(GO:0008083)[GSEA]


|  || Dataset | readcount.sample |
| Phenotype | sample.cls |
| Upregulated in class | TE1 |
| GeneSet | GROWTH\_FACTOR\_ACTIVITY(GO:0008083) |
| Enrichment Score (ES) | 0.5447271 |
| Normalized Enrichment Score (NES) | 1.7730771 |
| Nominal p-value | 0.0 |
| FDR q-value | 0.20989902 |
| FWER p-Value | 0.494 |
Table: GSEA Results Summary

  

Fig 1: Enrichment plot: GROWTH\_FACTOR\_ACTIVITY(GO:0008083)
  
Profile of the Running ES Score & Positions of GeneSet Members on the Rank Ordered List

  

| PROBE | DESCRIPTION   (from dataset) | GENE SYMBOL | GENE\_TITLE | RANK IN GENE LIST | RANK METRIC SCORE | RUNNING ES | CORE ENRICHMENT || 1 | 103243987 | BMP5 |  |  | 605 | 18.075 | 0.1670 | Yes |
| 2 | 103234997 | FGF21 |  |  | 635 | 10.068 | 0.2739 | Yes |
| 3 | 103246725 | BMP2 |  |  | 805 | 5.136 | 0.3215 | Yes |
| 4 | 103230001 | LOC103230001 |  |  | 876 | 4.440 | 0.3660 | Yes |
| 5 | 103215226 | FGF10 |  |  | 958 | 3.815 | 0.4034 | Yes |
| 6 | 103215153 | GDNF |  |  | 1149 | 3.171 | 0.4289 | Yes |
| 7 | 103220708 | GDF7 |  |  | 1668 | 2.296 | 0.4304 | Yes |
| 8 | 103218414 | FGF23 |  |  | 1727 | 2.267 | 0.4521 | Yes |
| 9 | 103236566 | VEGFC |  |  | 1999 | 2.084 | 0.4624 | Yes |
| 10 | 103234180 | GDF15 |  |  | 2279 | 1.945 | 0.4708 | Yes |
| 11 | 103221446 | VEGFA |  |  | 2567 | 1.833 | 0.4776 | Yes |
| 12 | 103237164 | GDF6 |  |  | 2906 | 1.731 | 0.4811 | Yes |
| 13 | 103238562 | INHBC |  |  | 3211 | 1.663 | 0.4853 | Yes |
| 14 | 103245650 | FGF7 |  |  | 3233 | 1.659 | 0.5022 | Yes |
| 15 | 103236710 | FGF2 |  |  | 3703 | 1.574 | 0.4981 | Yes |
| 16 | 103241402 | IL12A |  |  | 3747 | 1.567 | 0.5130 | Yes |
| 17 | 103241919 | FGF12 |  |  | 3757 | 1.564 | 0.5294 | Yes |
| 18 | 103226169 | INHBA |  |  | 3790 | 1.558 | 0.5447 | Yes |
| 19 | 103229341 | PGF |  |  | 6532 | 1.260 | 0.4356 | No |
| 20 | 103238487 | GDF11 |  |  | 7904 | 1.150 | 0.3866 | No |
| 21 | 103242230 | RABEP1 |  |  | 8377 | 1.127 | 0.3776 | No |
| 22 | 103235872 | BMP3 |  |  | 9252 | 1.069 | 0.3499 | No |
| 23 | 103235869 | FGF5 |  |  | 9261 | 1.068 | 0.3611 | No |
| 24 | 103238565 | INHBE |  |  | 9356 | 1.063 | 0.3683 | No |
| 25 | 103233641 | AMH |  |  | 9609 | 1.047 | 0.3682 | No |
| 26 | 103223339 | PDGFB |  |  | 10051 | 1.016 | 0.3594 | No |
| 27 | 103230082 | TGFB2 |  |  | 10295 | 1.004 | 0.3593 | No |
| 28 | 103232663 | FGF13 |  |  | 10526 | 0.989 | 0.3596 | No |
| 29 | 103242300 | FGF11 |  |  | 11896 | 0.900 | 0.3080 | No |
| 30 | 103244526 | GDF9 |  |  | 12006 | 0.894 | 0.3128 | No |
| 31 | 103246906 | PDGFA |  |  | 12033 | 0.892 | 0.3212 | No |
| 32 | 103234729 | TGFB1 |  |  | 12493 | 0.863 | 0.3099 | No |
| 33 | 103233749 | NRTN |  |  | 14182 | 0.758 | 0.2425 | No |
| 34 | 103233363 | VEGFB |  |  | 14651 | 0.730 | 0.2294 | No |
| 35 | 103231636 | VEGFD |  |  | 14720 | 0.726 | 0.2341 | No |
| 36 | 103233582 | FGF22 |  |  | 14945 | 0.711 | 0.2318 | No |
| 37 | 103224269 | CSF1 |  |  | 15636 | 0.666 | 0.2080 | No |
| 38 | 119621837 | GDF1 |  |  | 15847 | 0.651 | 0.2056 | No |
| 39 | 103222207 | BMP6 |  |  | 16936 | 0.574 | 0.1631 | No |
| 40 | 103236002 | LOC103236002 |  |  | 17074 | 0.566 | 0.1630 | No |
| 41 | 103243784 | BMP7 |  |  | 17287 | 0.550 | 0.1595 | No |
| 42 | 103229007 | BMP4 |  |  | 17488 | 0.533 | 0.1562 | No |
| 43 | 103229367 | TGFB3 |  |  | 17697 | 0.516 | 0.1525 | No |
| 44 | 103244512 | IL5 |  |  | 17718 | 0.514 | 0.1571 | No |
| 45 | 103224958 | ARTN |  |  | 17922 | 0.497 | 0.1533 | No |
| 46 | 103230288 | RABEP2 |  |  | 18427 | 0.447 | 0.1356 | No |
| 47 | 103244968 | FGF18 |  |  | 18446 | 0.445 | 0.1396 | No |
| 48 | 103236472 | MDK |  |  | 18705 | 0.421 | 0.1325 | No |
| 49 | 103225060 | BMP8B |  |  | 18737 | 0.417 | 0.1356 | No |
| 50 | 103229999 | LOC103229999 |  |  | 19125 | 0.378 | 0.1224 | No |
| 51 | 103216911 | INHBB |  |  | 19298 | 0.362 | 0.1185 | No |
| 52 | 103244503 | CSF2 |  |  | 19480 | 0.341 | 0.1141 | No |
| 53 | 103217920 | INHA |  |  | 19532 | 0.336 | 0.1154 | No |
| 54 | 103225049 | BMP8A |  |  | 19704 | 0.316 | 0.1112 | No |
| 55 | 103246343 | FGF19 |  |  | 19977 | 0.276 | 0.1020 | No |
| 56 | 103237005 | IL7 |  |  | 20062 | 0.263 | 0.1010 | No |
| 57 | 103248989 | FGF9 |  |  | 20429 | 0.206 | 0.0869 | No |
| 58 | 103244714 | FGF1 |  |  | 20803 | 0.128 | 0.0716 | No |
Table: GSEA details
[plain text format]

  

Fig 2: GROWTH\_FACTOR\_ACTIVITY(GO:0008083)
  
Blue-Pink O' Gram in the Space of the Analyzed GeneSet

  

Fig 3: GROWTH\_FACTOR\_ACTIVITY(GO:0008083): Random ES distribution
  
Gene set null distribution of ES for
 **GROWTH\_FACTOR\_ACTIVITY(GO:0008083)**
